# Sciatica due to Schwannoma at the Sciatic Notch

**DOI:** 10.1155/2013/510901

**Published:** 2013-05-19

**Authors:** Yavuz Haspolat, Feyza Unlu Ozkan, Ismail Turkmen, Bahattin Kemah, Yalcin Turhan, Serhan Sarar, Korhan Ozkan

**Affiliations:** ^1^Department of Plastic and Reconstructive Surgery, Istanbul Medeniyet University Goztepe Training and Research Hospital, 34732 Istanbul, Turkey; ^2^Department of Physical Therapy and Rehabilitation, Istanbul Fatih Sultan Mehmet Training and Research Hospital, Istanbul, Turkey; ^3^Department of Orthopaedics and Traumatology, Istanbul Medeniyet University Goztepe Training and Research Hospital, 34732 Istanbul, Turkey; ^4^Department of Orthopaedics and Traumatology, Duzce State Hospital, 81020 Duzce, Turkey; ^5^Department of Anesthesiology and Reanimation, Istanbul Medeniyet University Goztepe Training and Research Hospital, 34732 Istanbul, Turkey; ^6^Department of Orthopaedics and Traumatology, Faculty of Medicine, Istanbul Medeniyet University, 34732 Istanbul, Turkey

## Abstract

Schwannomas are rarely seen on the sciatic nerve and can cause sciatica. In this case report we aimed to present an unusual location of schwannoma along sciatic nerve that causes sciatica. A 60-years-old-man was admitted to us with complaints of pain on his thigh and paresthesia on his foot. Radiography of the patient revealed a solitary lesion on the sciatic nerve. The lesion was excised and the symptoms resolved after surgery.

## 1. Introduction

Schwannomas are derived from Schwann cells of neuroectoderm. They serve for the formation of myelin sheaths of nerves that insulate nerve and facilitate the transmission of an impulse [1]. It is a benign encapsulated slow-growing tumor. Unlike neurofibromas, schwannomas do not transverse through the nerve but remain in the sheath lying on top of the nerve. 

Sciatica is defined as pain along the course of the sciatic nerve and its branches. Characteristically the patients report gluteal pain radiating down the posterior thigh and leg with paresthesia in the calf and foot along the route of the sciatic nerve [2]. 

In this report we aimed to present a patient with the symptoms of sciatica for five years due to unrecognized eight-centimetre schwannoma of sciatic nerve at the sciatic notch of pelvis. 

## 2. Case Presentation

**Figure 1:**
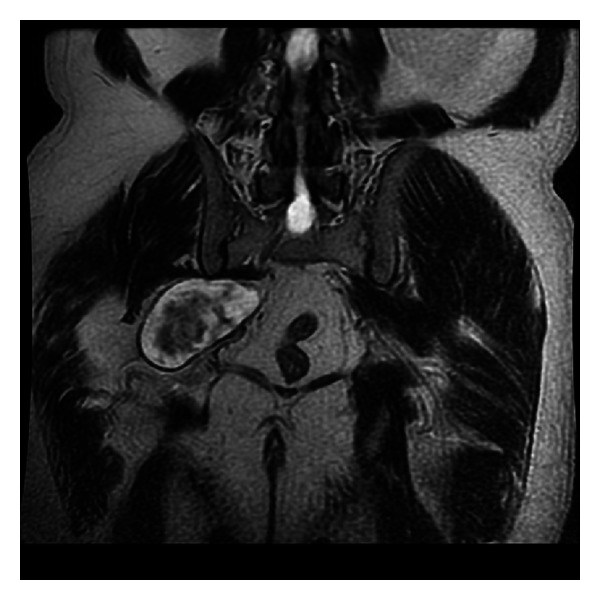
Coronal MRI of the lesion.

A 60-years-old patient with long standing symptoms of pain and paresthesia was referred to algology and physical therapy departments for finding out the possible etiology as his symptoms of sciatica increased. His past medical history revealed that he had been treated with the diagnosis of lumbosacral degenerative pathology for a long period of time although he had irrelevant lumbosacral magnetic resonance imaging (MRI). He had been further investigated with electromyography (EMG) that revealed decrease peroneal and tibial motor and sensory nerve conduction velocity. Sural and superficial peroneal sensory action potentials were decreased. Pelvic MRI was taken for the possible lesion compressing the sciatic nerve which displayed the noncontrast enhancing mass on the sciatic nerve at the sciaitc notch section (Figure 1).

**Figure 2:**
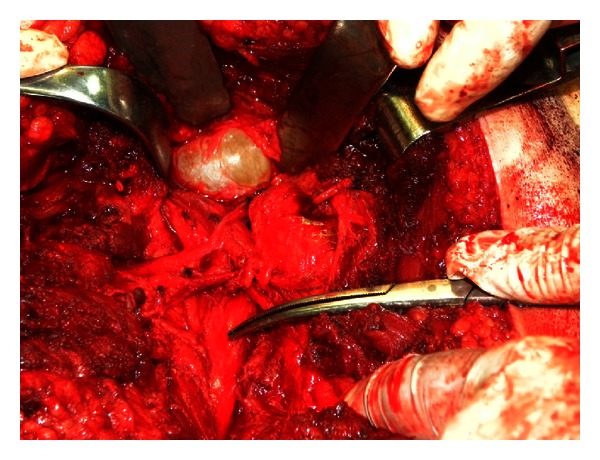
Intraoperative photograph of the lesion. Clamp shows sciatic nerve.

**Figure 3:**
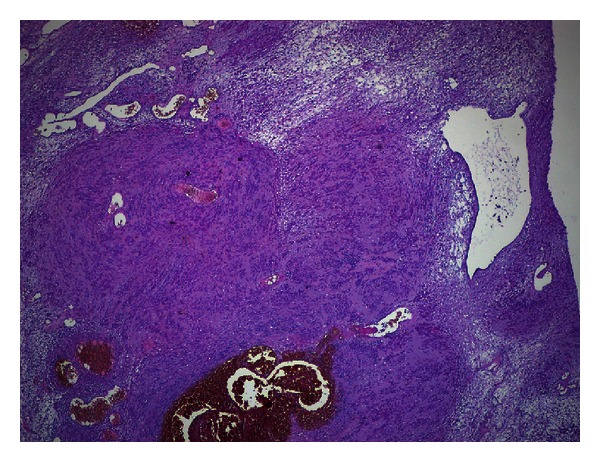
Microscopic photograph of the lesion reveals ancient form schwannoma. Hyaline degeneration and edema on the ground. Undulant and hypocellular Schwann cells are placed as clusters.

Surgery was planned with the possible diagnosis of schwannoma or neurofibroma. An incision was made through the route of sciatic nerve and the nerve was explored till the sciatic notch beginning proximally. The soft tissue mass on the sciatic nerve was seen and removed from the nerve sheath (Figure 2). The pathological examination result was consistent with the diagnosis of schwannoma (Figure 3). The patient's symptoms resolved 3 weeks after the surgery. 

## 3. Discussion

Sciatica is most commonly caused by herniated disc compressing the nerve roots or lumbosacral degenerative pathology, although very infrequent entrapment of sciatic nerve along its course within the pelvis or the lower extremity due to heterotopic ossification, misplaced intramuscular injections, myofascial bands in the thigh, myositis ossificans of biceps muscle, posttraumatic or anticoagulant-induced hematomas, compartment syndrome, and bone and soft tissue tumors can cause sciatica [3–5]. 

Schwannomas are common, slow-growing benign tumors of the sheath of peripheral nerves arising from the schwann cells and involvement of sciatic nerve is very rare. Most are solitary lesions and multiplicity or malignant transformation occurs very rarely [6, 7]. Although few cases of schwannoma along the sciatic nerve were published in the English literature this is the first case of sciatic nerve compression at the level of sciatic notch due to schwannoma [8, 9]. 

 Pain not responding to rest or activity, sensory and motor dysfunction along the nerve distribution are the most common manifestations. Surgical intervention should be the treatment of choice in order to prevent neurological deficits and exclude the possibility of malignancy. 

Sciatic nerve schwannomas should be kept in mind as a causative factor of sciatica and magnetic resonance imaging of the sciatic nerve in case of suspicion especially in patients with irrelevant lumbosacral MRI is important for diagnosis.

## References

[1] Rustagi T, Badve S, Parekh AN (2012). Sciatica from a foraminal lumbar root schwannoma: case report and review of literature. *Case Reports in Orthopedics*.

[2] Valat JP, Genevay S, Marty M, Rozenberg S, Koes B (2010). Sciaitica. *Best Practice & Research Clinical Rheumatology*.

[3] Mendeszoon MJ, Cunningham N, Crockett RS Schwannoma: a case report. *The Foot and Ankle Online Journal*.

[4] Bickels J, Kahanovitz N, Rubert CK (1999). Extraspinal bone and soft-tissue tumors as a cause of sciatica: clinical diagnosis and recommendations: analysis of 32 cases. *Spine*.

[5] Feinberg J, Sethi S (2006). Sciatic neuropathy: case report and discussion of the literature on postoperative sciatic neuropathy and sciatic nerve tumors. *HSS Journal*.

[6] Woodruff JM, Selig AM, Crowley K, Allen PW (1994). Schwannoma (neurilemoma) with malignant transformation: a rare, distinctive peripheral nerve tumor. *American Journal of Surgical Pathology*.

[7] Sehgal VN, Gupta RL, Bhatia A, Kumar S, Jain S, Kapoor V (1999). Solitary cellular schwannoma (neurilemmoma) showing malignant changes: evaluation through magnetic resonance imaging (M.R.I.), surgical intervention, and histopathology. *Journal of Dermatology*.

[8] Gominak SC, Ochoa JL (1998). Sciatic schwannoma of the thigh causing foot pain mimicking plantar neuropathy. *Muscle Nerve*.

[9] Yamamoto T, Maruyama S, Mizuno K (2001). Schwannomatosis of the sciatic nerve. *Skeletal Radiology*.

